# Supplementary material for: Common and distinct neurofunctional signatures of dynamic naturalistic emotion regulation strategies
Source: Nat Commun. 2026 Mar 17;17:4272. doi: 10.1038/s41467-026-70708-5 (PMC13168294; doi:10.1038/s41467-026-70708-5)
Supplement: Supplementary file 1 — Supplementary Information [file 41467_2026_70708_MOESM1_ESM.pdf]

Supplementary Information

Common and distinct neurofunctional signatures of dynamic naturalistic emotion regulation strategies

Authors

Heng Jiang (姜恒), Jingxian He (何婧娴), Kaeli Zimmermann, Xinqi Zhou (周鑫岐), Xianyang Gan (甘鲜阳), Stefania Ferraro, Lan Wang (王岚), Bo Zhou (周波), Liyuan Li (李立圆), Keith M Kendrick [1, 2], Weihua Zhao (赵伟华), Dezhong Yao (尧德中), Tifei Yuan (袁逖飞), Feng Zhou (周峰)*, Benjamin Becker*

This file includes:

Subjective negativity rating in the discovery and validation cohort......................2

MRI data acquisition and preprocessing of the clinical application cohort...........3

Supplementary Tables...6

Supplementary Figures 14

References..23

Subjective negativity rating in the discovery and validation cohort

In the discovery cohort (n=59, utilized to develop the decoders) a one factorial repeated-measures analysis of variance (ANOVA) with the factor condition (NV, NeutV) revealed a significant main effect (F [1, 58]=1039.434, P=9.96×10^{-39}, η_p^2=0.947; Fig.2a). Further simple effects analysis revealed that the negative view (NV) condition (6.52±1.21, Mean±SD) induced significantly higher negative emotional experience than the neutral view (NeutV) condition (1.58±0.55, P=9.96×10^{-39}, 95% confidence interval (CI) [4.63, 5.25]). Given that the assumption of sphericity for one factor of the repeated-measures ANOVA of strategies (NV, negative–acceptance (NA), negative–reappraisal (NR)) was violated, the Greenhouse-Geisser correction was used, revealing a significant main effect (F [2, 95]=81.668, P=3.18×10^{-19}, η_p^2=0.585). Further simple effects analysis revealed that both reappraisal (4.54±1.58) and acceptance (5.44±1.53) lead to significantly reduced subjective negative experience (NV: 6.52±1.21; NR vs. NV, P=1.54×10^{-7}, 95% CI [-2.40, -1.55]; NA vs. NV, P=4.40×10^{-16}, 95% CI [-1.49, -0.65]), indicating that both strategies allowed the participants to successfully regulate their negative emotional state. Reappraisal results in lower negative feelings than acceptance (P=2.02×10^{-10}, 95% CI [-1.18, -0.62]). The successful emotion regulation was also mirrored in the self-report success rate of emotion regulation (9 - very successful, 1 - not successful at all, averaged success rating: discovery cohort: 7.51±1.01, validation cohort: 7.53±0.98).

In the validation cohort (n=33), one factor (stimulus type: NV, UV) repeated-measures ANOVA revealed a significant main effect (F [1, 32]=255.626, P=8.15×10^{-17}, η_p^2=0.947). Further simple effects analysis revealed that the negative clips induced significantly stronger negative emotions than the neutral clips (NV vs. NeutV, 6.74±1.44 vs. 1.86±1.02, P=8.15×10^{-17}, 95% CI [4.26, 5.50]). The assumption of sphericity from one factor repeated-measures ANOVA of strategies (NV, NA, NR) was violated, so the Greenhouse-Geisser correction was used, revealing a significant main effect (F [2, 52]=56.637, P=1.32×10^{-12}, η_p^2=0.639). Both reappraisal and acceptance

lead to significantly reduced subjective negative emotion (NR vs. NV, (4.43±1.83) vs. (6.74±1.44), $P=7.07\times10^{-7}$, 95% CI [-2.95, -1.66]; NA vs. NV, (5.3±1.53) vs. (6.74±1.44), $P=1\times10^{-6}$, 95% CI [-2.02, -0.86]). Reappraisal results in greater reduction than acceptance ($P=2.4\times10^{-5}$, 95% CI [-1.27, -0.45]).

MRI data acquisition and preprocessing of the clinical application cohort

Participants

Data from eighteen male healthy control (HC) participants and twenty-three male heavy recreational cannabis users (CU) were obtained from our previous study[1]. For the present study, we increased the sample size and recruited thirty-six healthy male HC participants and thirty-six male CU participants according to comparable criteria to our previous study[1].

For all recruited participants, exclusion criteria included (1) a history of psychiatric disorder (measured by the Mini-International Neuropsychiatric Interview, M.I.N.I.[2]), except for cannabis use disorder, (2) regular/current use of psychoactive/cardiovascular medication, (3) positive urine screen for the substances cocaine, methamphetamine, amphetamine, or methadone, or (4) breath alcohol level >0.00. For CU, additional inclusion criteria included (1) long-term regular cannabis use (use on over 200 occasions, in the new sample cannabis users had used cannabis regularly for 77.90±51.53 months; during the past year on 25.35±6.37 days per month) additional exclusion criteria included (1) reported having used other illicit substances on > 50-lifetime occasions (2) use of cannabis or other substances in the 24 hours before the experiment. For HC, additional exclusion criteria included a positive of tetrahydrocannabinol (THC) testing on the day of the fMRI scanning.

Three HC and seven CU newly collected participants were excluded due to excessive head motion (> 3 mm or 3°); three HC and three CU were excluded due to being detected as outliers based on their emotion regulation success (defined as the mean decrease of the negative affect rating for reappraisal (“distance”) trials relative to

ratings for emotional reactivity (“spontaneous_negative”)[1]. Thirty HC (26.1 ± 4.8 years old) and twenty-six CU newly recruited participants (26.6 ± 5.8 years old) were included in the final analysis, leading to a total of 48 HC and 49 CU participants were included as the clinical application cohort. No significant group difference in years of age and education. All participants provided written informed consent.

Stimuli and Paradigm

The stimuli and paradigm are similar to our previous study[1]. Briefly, The paradigm included a total of 90 images from the International Affective Picture System[3] (IAPS, National Institute of Mental Health Center for Emotion and Attention, University of Florida), consisting of 30 neutral and 60 negative images. The negative images were divided into two sets: 30 for the spontaneous viewing condition (“Spontaneous” - “Spontan”) and 30 for the distancing (reappraisal) condition (“Distance” - “Distanz”).

Each trial began with a 5–7 s jittered fixation cross, followed by a 2 s instruction screen indicating the required condition (“Spontan” for passive viewing of negative or neutral images, or “Distanz” for active distancing during negative images). After a 2–4 s jittered interstimulus interval (ISI), the target image was presented for 8 s, during which participants either viewed the image naturally (“Spontan”) or applied the distancing strategy (“Distanz”). Each trial concluded with a 2–4 s ISI and a subsequent self-report rating of negative affect on a 0–100 scale (0 = no negative affect, 50=moderate negative affect, 100=strong negative affect). Stimulus presentation and behavioral responses were recorded using E-Prime software (Psychology Software Tools, Inc.).

Participants were trained prior to scanning: for the spontaneous condition, they were instructed to respond naturally without regulating their emotions; for the distancing condition, they were instructed to reduce negative affect by mentally distancing themselves from the image, for example, by viewing the scene as an uninvolved observer. Participants also described the strategy in their own words and provided examples of implementation before starting the task.

MRI data acquisition and preprocessing

MRI data were acquired using an identical scanner and sequence settings to our previous study[1]. Data were collected on a Siemens Trio 3T MRI scanner (Siemens, Erlangen, Germany). Functional images were acquired using a T2*-weighted echo-planar imaging (EPI) BOLD sequence with the following parameters: repetition time (TR)=2500 ms, echo time (TE)=30 ms, 37 slices, voxel size=2×2×3 mm^3, flip angle=90°, and field of view (FOV)=192 mm. A high-resolution T1-weighted structural scan was also acquired (TR=1660 ms, TE=2.54 ms, 208 slices, voxel size=0.8×0.8×0.8 mm^3, FOV=256 mm) to screen for structural abnormalities and facilitate normalization of the functional data. Use the same preprocessing script as we did on the discovery and validation cohort (only modifying the parameters based on the sequence setting).

Supplementary Tables

Table S1. Spatial similarity between model encoding and ROIs, as well as functional networks

Name	Cosine Similarity		
	NERS-A	NERS-R	NNES
Prefrontal ROIs			
dlPFC	0.097	0.2216	0.0535
vlPFC	0.0761	0.0874	0.0539
dmPFC	0.1542	0.2251	0.009
vmPFC	0.0895	0.1494	0.0034
OFC	0.0471	0.1781	0
ACC	0.1034	0.0852	0.0244
Functional networks			
Visual	0.4929	0.5694	0.4974
Somatomotor	0.4863	0.0419	0.1964
dAttention	0.3239	0.1412	0.5498
vAttention	0.2288	0.0572	0.2863
Limbic	0.1812	0.1531	0.0294
Frontoparietal	0.1267	0.2713	0.1104
Default	0.4282	0.5837	0.0807

NERS-A, naturalistic emotion regulation signature – acceptance; NERS-R, naturalistic emotion regulation signature – reappraisal; NNES, naturalistic negative emotion signature; dlPFC, dorsolateral prefrontal cortex; vlPFC, ventrolateral prefrontal cortex; dmPFC, dorsomedial prefrontal cortex; vmPFC, ventromedial prefrontal cortex; OFC, orbitofrontal cortex; ACC, anterior cingulate cortex; vAttention, ventral attention; dAttention, dorsal attention.

Table S2. Classification performance between decoders on the validation cohort

Condition	Decoder	ACC (%)	SD	Sens (%)	CI	Spec (%)	CI	Effect size	*P*
Validation cohort (n=33)									
NV vs. NeutV	NNES	100	0	100	100–100	100	100–100	3.55	**2.33×10^{-10}**
	PINES	91	5	91	80–100	91	79–100	1.16	**1.40×10^{-6}**
	NERS-A	70	8	70	53–85	70	53–85	0.95	**0.035**
	NERS-R	3	3	3	0–10	3	0–10	-33.03	**7.92×10^{-9}**
NA vs. NV	NNES	48	8.7	48	31–66	48	32–67	-0.3	1
	PINES	36	8.4	36	20–54	36	21–53	-0.12	0.1628
	NERS-A	70	8	70	53–86	70	53–85	0.47	**0.0351**
	NERS-R	61	8.5	36	19–53	36	21–53	0.61	0.2962
NR vs. NV	NNES	15	6.2	15	3–29	15	3–28	-0.95	**6.60×10^{-5}**
	PINES	45	8.7	45	30–63	45	29–63	-0.02	0.7283
	NERS-A	70	8	70	54–85	70	53–85	0.72	**0.0351**
	NERS-R	88	5.7	88	75–97	88	76–97	1.42	**1.10×10^{-5}**

Statistics performance was evaluated using a two-alternative forced-choice procedure and significance was assessed using a one-sided binomial test comparing accuracy against the chance level of 0.5. No multiple-comparison correction was applied. ACC, accuracy; Sens, sensitivity; specificity; SD, standard deviation; CI, confidence interval; NV, view (react to) negative clips; NeutV, view (react to) neutral clips; NA, use acceptance strategy to negative clips; NR, use reappraisal strategy to negative clips; NNES, naturalistic negative emotion signature; PINES, picture-induced negative emotion signature; NERS-A, naturalistic emotion regulation signature – acceptance; NERS-R, naturalistic emotion regulation signature – reappraisal. Bold indicates $P<0.05$.

Table S3. Prediction-outcome accuracy (mean ± std) of distinguishing between NA vs. NV with various numbers of voxels (for the Discovery Cohort)

Number of voxels	Vis	SM	dA	vA	Limb	FP	DMN	PF	WB
**50**	58.83	56.88*	59.13	55.06*	55.2*	57.99*	58.47	58.24*	58.73
	3.56	3.52	3.58	3.38	3.56	3.68	3.68	3.67	3.93
**150**	61.95	58.81*	61.86	54.99*	54.93*	58.57*	60.37*	59.32*	62.18
	3.19	3.31	3.21	2.88	3.16	3.04	3.27	3.29*	3.41
**250**	62.84*	59.12*	62.55*	55.24*	55.42*	59.18*	61.51*	59.82*	65.17
	2.96	2.99	2.75	2.76	2.83	2.79	2.99	2.86	3.4
**500**	62.71*	59.41*	63.74*	55.95*	56.18*	60.15*	62.7*	60.58*	68.91
	2.62	2.61	2.44	2.53	2.35	2.57	2.85	2.71	3.18
**750**	62.15*	60.15*	64.48*	56.36*	56.44*	60.42*	63.27*	61.01*	70.91
	2.36	2.36	2.4	2.51	2.17	2.34	2.75	2.49	3.06
**1000**	61.78*	60.69*	64.72*	56.65*	56.63*	60.46*	63.55*	61.26*	72.27
	2.27	2.17	2.22	2.27	2.09	2.23	2.55	2.31	2.93
**2000**	61.09*	61.68*	65.32*	56.95*	56.86*	60.71*	64.04*	61.8*	74.6
	1.86	2.02	1.88	2.08	1.7	1.87	2.23	2.01	2.5
**4000**	60.57*	62.35*	65.71*	56.94*	56.79*	61.14*	64.63*	62.28*	76.03
	1.68	1.78	1.62	1.73	1.35	1.36	2.02	1.66	2.15
**6000**	60.38*	62.65*	65.99*	57.07*	56.66*	61.21*	64.94*	62.59*	76.43
	1.54	1.65	1.37	1.46	0.99	1.15	1.9	1.49	1.99
**8000**	60.31*	62.89*	66.05*	57.28*	56.42*	61.3*	65.1*	62.85*	76.54
	1.47	1.47	1.18	1.31	0.68	0.97	1.82	1.31	1.68
**10000**	60.23*	63.01*	66.07*	57.47*	NA	61.34*	65.2*	62.99*	76.81
	1.32	1.39	1.03	1.17	NA	0.83	1.73	1.17	1.58
**14000**	60.05*	63.39*	65.93*	NA	NA	61.3*	65.39*	63.22*	76.94
	1.07	1.18	0.62	NA	NA	0.64	1.5	0.95	1.46

**18000**	59.72*	63.67*	NA	NA	NA	61.5*	65.55*	63.51*	77.02
	0.71	0.78	NA	NA	NA	0.47	1.37	0.75	1.46
**25000**	NA	NA	NA	NA	NA	NA	65.73*	63.94*	77.12
	NA	NA	NA	NA	NA	NA	0.99	0.55	1.3
**Full**	59.32	63.56	65.25	57.63	56.78	61.86	65.25	64.41	77.97

Vis, visual network; SM, somatomotor network; dA, dorsal attention network; vA, ventral attention network; Limb, limbic network; FP, frontoparietal network; DMN, default mode network; PF, prefrontal cortex; NA, not applicable. * and # indicate that the prediction is significantly lower or higher as compared with the whole-brain model using the same number of voxels, separately (two-tailed t-test; $P<0.05$, Bonferroni corrected).

Table S4. Prediction-outcome accuracy (mean ± std) of distinguishing between NR vs. NV with various numbers of voxels (for the Discovery Cohort)

Number of voxels	Vis	SM	dA	vA	Limb	FP	DMN	PF	WB
**50**	64.64	61.1*	65.25#	62.26*	57.63*	61.67*	66.07#	61.09*	64.42
	4.41	3.65	4.17	4.1	3.91	4.22	4.41	4.04	5.05
**150**	69.5#	60.54*	68.02*	64.06*	57.89*	63.41*	67.38*	62.23*	68.82
	3.55	3.17	3.4	3.46	3.33	3.36	3.46	3.4	4.32
**250**	71.02*	60.67*	70.34*	65.76*	57.67*	64.65*	69.06*	63.01*	72.02
	3.22*	2.91	3.15	3.44	2.95	3.18	3.1	3.07	3.83
**500**	71.47*	61.07*	72.84*	68.36*	57.85*	66.46*	70.8*	64.27*	74.88
	2.63	2.68	2.73	3.13	2.61	2.84	2.75	2.74	3.46
**750**	71.58*	61.54*	73.7*	69.49*	58.08*	67.11*	71.45*	64.58*	76.53
	2.44	2.48	2.43	2.85	2.47	2.54	2.52	2.45	2.9
**1000**	71.52*	61.73*	74.14*	70.22*	58.21*	67.43*	71.95*	64.62*	77.22
	2.23	2.33	2.22	2.69	2.39	2.44	2.4	2.24	2.69
**2000**	71.2*	62.2*	75.04*	71.56*	58.45*	67.86*	72.82*	64.71*	78.68
	1.89	1.91	1.74	2.13	1.98	1.93	2.07	1.98	2.09
**4000**	70.84*	62.66*	75.45*	72.61*	58.46*	68.02*	73.35*	65.06*	79.59
	1.58	1.49	1.34	1.65	1.64	1.53	1.7	1.72	1.88
**6000**	70.65*	62.73*	75.68*	73.19*	58.3*	68.03*	73.57*	65.23*	79.92
	1.39	1.31	1.05	1.33	1.36	1.39	1.55	1.5	1.67
**8000**	70.59*	62.81*	75.76*	73.62*	57.99*	68.07*	73.68*	65.38*	80.14
	1.24	1.18	0.93	1.16	1	1.21	1.42	1.39	1.56
**10000**	70.51*	62.9*	75.91*	73.94*	NA	68.2*	73.71*	65.6*	80.35
	1.19	1	0.76	0.94	NA	1.12	1.32	1.26	1.58
**14000**	70.47*	62.93*	76.14*	NA	NA	68.45*	73.78*	65.8*	80.73
	1.01	0.8	0.35	NA	NA	0.85	1.15	1.09	1.44

**18000**	70.46*	62.93*	NA	NA	NA	68.85*	73.76*	65.88*	80.94
	0.83	0.55	NA	NA	NA	0.63	1.02	0.94	1.36
**25000**	NA	NA	NA	NA	NA	NA	73.55*	65.98*	81.28
	NA	NA	NA	NA	NA	NA	0.7	0.68	1.23
**Full**	70.34	62.71	76.27	74.58	58.47	69.49	72.88	66.1	83.05

Vis, visual network; SM, somatomotor network; dA, dorsal attention network; vA, ventral attention network; Limb, limbic network; FP, frontoparietal network; DMN, default mode network; PF, prefrontal cortex; NA, not applicable. * and # indicate that the prediction is significantly lower or higher as compared with the whole-brain model using the same number of voxels, separately (two-tailed t-test; $P<0.05$, Bonferroni corrected).

Table S5. Prediction-outcome accuracy (mean ± std) of distinguishing between NV vs. NeutV with various numbers of voxels (for the Discovery Cohort)

Number of voxels	Vis	SM	dA	vA	Limb	FP	DMN	PF	WB
**50**	73.54*	81.37$^{\#}$	79.5$^{\#}$	80.07$^{\#}$	62.15*	74.11*	70.12*	72.99*	77.96
	4.47	4.56	4.24	4.49	3.97	4.41	4.16	4.13	4.95
**150**	80.56*	86.1$^{\#}$	84.39	85.18$^{\#}$	63.27*	77.57*	71.65*	74.89*	84.47
	3.74	3.38	3.23	3.49	3.46	3.47	3.32	3.27	3.87
**250**	82.61*	87.84$^{\#}$	86.67	87.3$^{\#}$	63.95*	79.31*	73.36*	75.99*	86.91
	3.12	2.82	2.61	2.85	3	3.11	3.04	3.04	3.16
**500**	84.14*	89.19*	88.41*	89.2*	64.44*	81.34*	75.27*	77.35*	89.74
	2.45	2.21	2.03	2.3	2.51	2.7	2.57	2.6	2.97
**750**	84.65*	89.7*	88.92*	89.97*	64.58*	82.06*	76.09	78.04*	90.96
	2.25	1.95	1.82	2.09	2.23	2.34	2.2	2.26	2.35
**1000**	84.8*	89.89*	89.02*	90.32*	64.63*	82.51*	76.36	78.31*	91.47
	2.1	1.82	1.71	1.99	2.06	2.17	2.02	2.2	2.14
**2000**	84.86*	90.65*	89.37*	90.98*	64.58*	83.26*	76.79*	78.65*	92.77
	1.87	1.52	1.41	1.63	1.65	1.53	1.51	1.85	1.63
**4000**	84.6*	91.2*	89.45*	91.14*	64.42*	83.65*	76.91*	78.88*	93.35
	1.63	1.2	1.15	1.34	1.37	1.16	1.12	1.58	1.37
**6000**	84.41*	91.51*	89.6*	91.22*	64.21*	83.79*	76.84*	78.96*	93.69
	1.44	1.13	1.01	1.12	1	0.96	1	1.48	1.24
**8000**	84.24*	91.81*	89.66*	91.13*	63.86*	83.84*	76.75*	79.02*	93.93
	1.3	1.04	0.91	0.97	0.48	0.74	0.87	1.33	1.11
**10000**	84.12*	92.03*	89.75*	91.01*	NA	83.89*	76.66*	79.07*	93.89
	1.14	0.92	0.79	0.84	NA	0.59	0.82	1.24	1.08
**14000**	83.96*	92.33*	89.93*	NA	NA	83.9*	76.53*	79.07*	94.01
	0.9	0.75	0.41	NA	NA	0.27	0.79	1.12	1.08

**18000**	83.74*	92.66*	NA	NA	NA	83.9*	76.37*	79.07*	94.17
	0.71	0.62	NA	NA	NA	0	0.73	0.99	1.09
**25000**	NA	NA	NA	NA	NA	NA	76.03*	78.86*	94.11
	NA	NA	NA	NA	NA	NA	0.57	0.72	0.94
**Full**	83.05	93.22	89.83	90.68	63.56	83.9	75.42	78.81	94.07

Vis, visual network; SM, somatomotor network; dA, dorsal attention network; vA, ventral attention network; Limb, limbic network; FP, frontoparietal network; DMN, default mode network; PF, prefrontal cortex; NA, not applicable. * and # indicate that the prediction is significantly lower or higher as compared with the whole-brain model using the same number of voxels, separately (two-tailed t-test; $P<0.05$, Bonferroni corrected).

Supplementary Figures

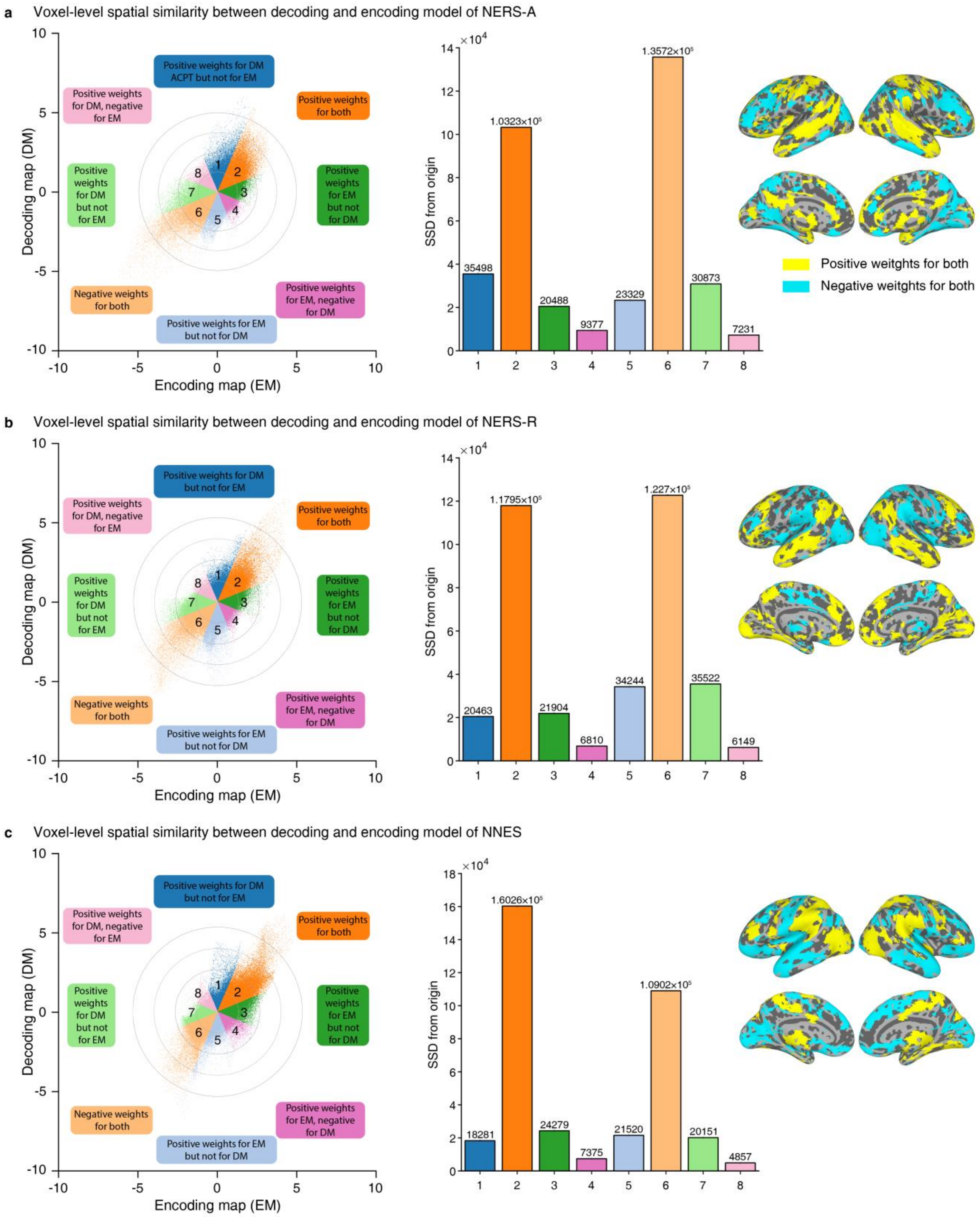

Fig.S1 | Voxel-level spatial similarity between decoding and encoding models.

Voxel-level spatial similarity between the normalized unthresholded decoding weighted

map and encoding map of NERS-A (**a**), NERS-R (**b**), and NNES (**c**), separately. Scatter plots illustrate normalized unthresholded voxel beta weights of the decoding map (y-axis) and encoding map (x-axis). Colored octants indicate voxels of shared positive (Octants 2) or shared negative (Octants 6), selective positive weights for decoding map (Octant 1) or encoding map (Octant 3), selective negative weights for decoding map (Octant 5) or encoding map (Octant 7), and voxel weights opposite for the two maps (Octants 4 and 8). The middle bars indicate the sum of squared distances from the origin (0, 0) for each octant, which integrates the number of voxels and combined weights. Displayed on the right are the voxel distributions of the patterns with consistent positive correlations, i.e., voxels originating from the shared positive (Octants 2, shown in yellow) and shared negative (Octants 6, shown in blue) sectors. The numerical values shown at the top of each bar represent these total squared distances for the corresponding octant. The resulting spatial patterns are largely consistent with and cover a broader spatial extent than those identified by the FDR-corrected Haufe transformation.

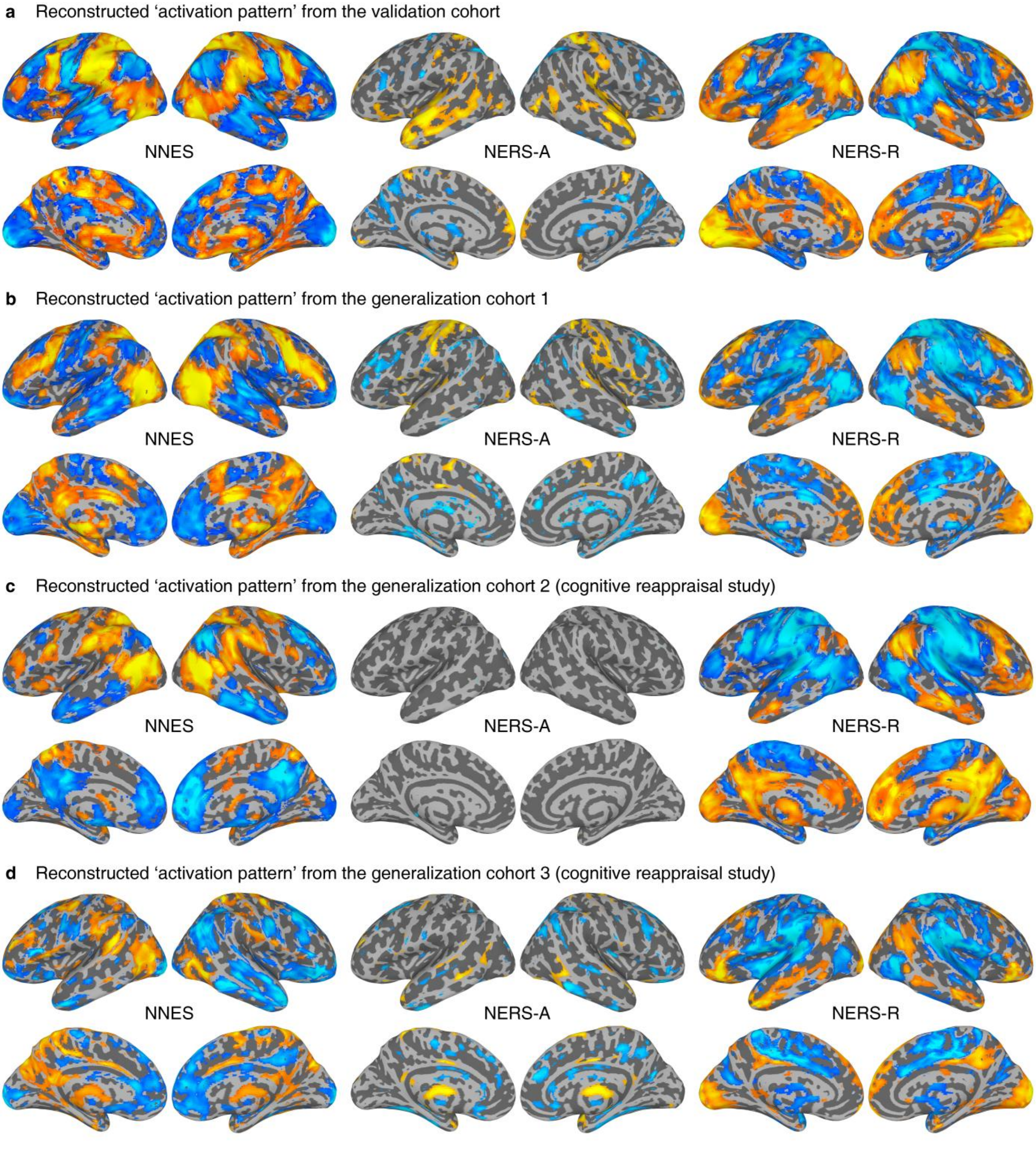

Fig.S2 | Reconstructed 'activation pattern' from the validation and generalization cohorts. FDR corrected (q<0.05) group-level reconstructed 'activation pattern' transformed from the validation cohort (**a**), generalization cohort 1 (**b**), generalization cohort 2 (**c**), and generalization cohort 3 (**d**). The color indicates the direction of the relationship between each voxel and the target variable – i.e., which

voxels are positively (red) or negatively (blue) related to the corresponding mental processing (e.g., emotion regulation strategies). Consistent reconstructed activation patterns were observed in datasets where the decoders achieved significantly accurate predictions (e.g., NERS-R across all datasets), indicating the robustness of the findings. Some inconsistencies were noted, for example, in generalization cohorts 2 and 3 for NERS-A, possibly because the reconstructed 'activation patterns' were derived from data corresponding to a different mental process than that used to train the decoder (i.e., reappraisal rather than acceptance).

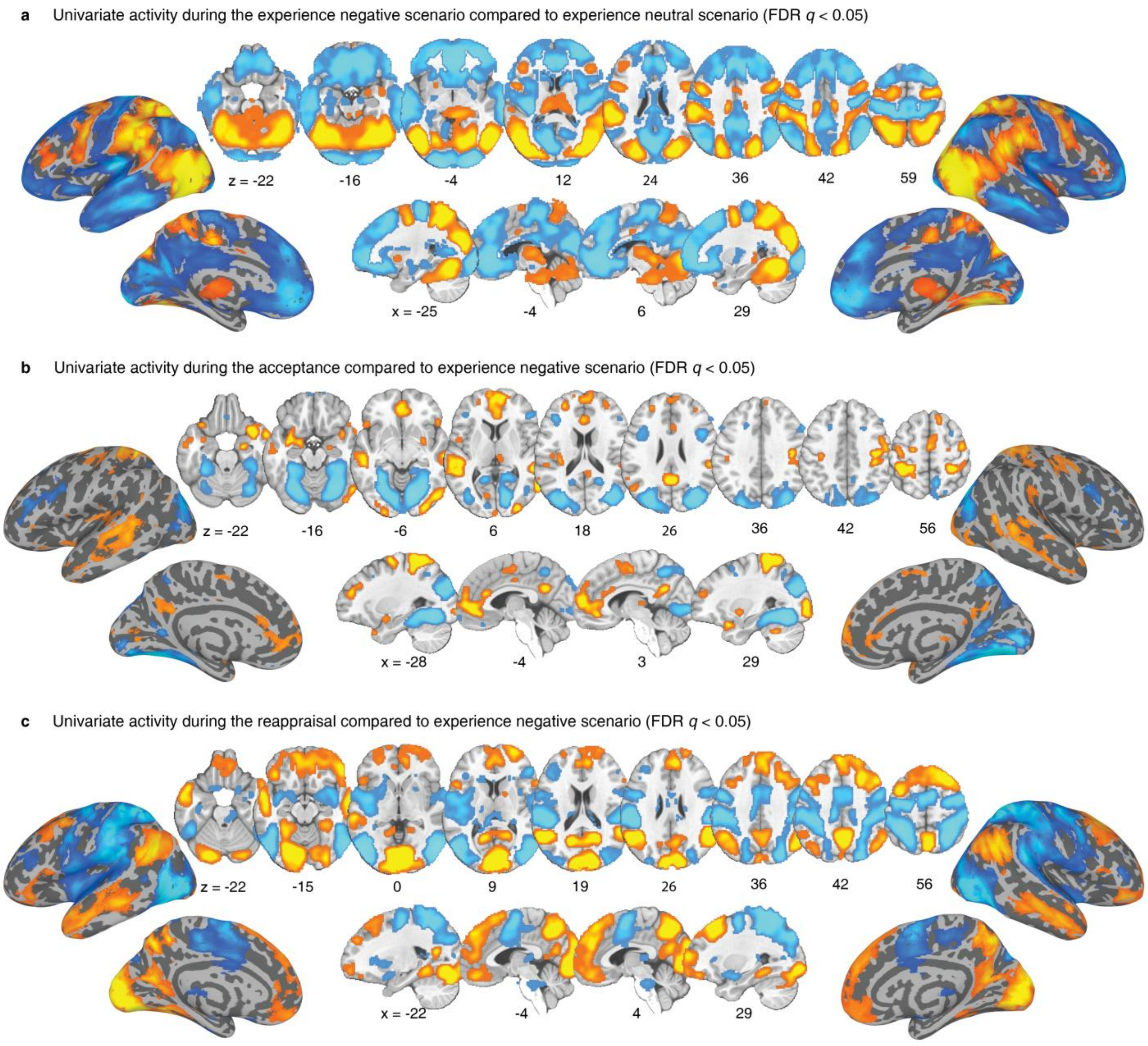

Fig.S3 | Brain activation from mass-univariate analysis. Contrast images representing the response to naturalistic negative scenario (view negative scenario (NV) vs. view neutral scenario (NeutV) (**a**), accept negative scenario (NA) vs. NV (**b**), and reappraisal negative scenario (NR) vs. NV (**c**) (FDR q<0.05).

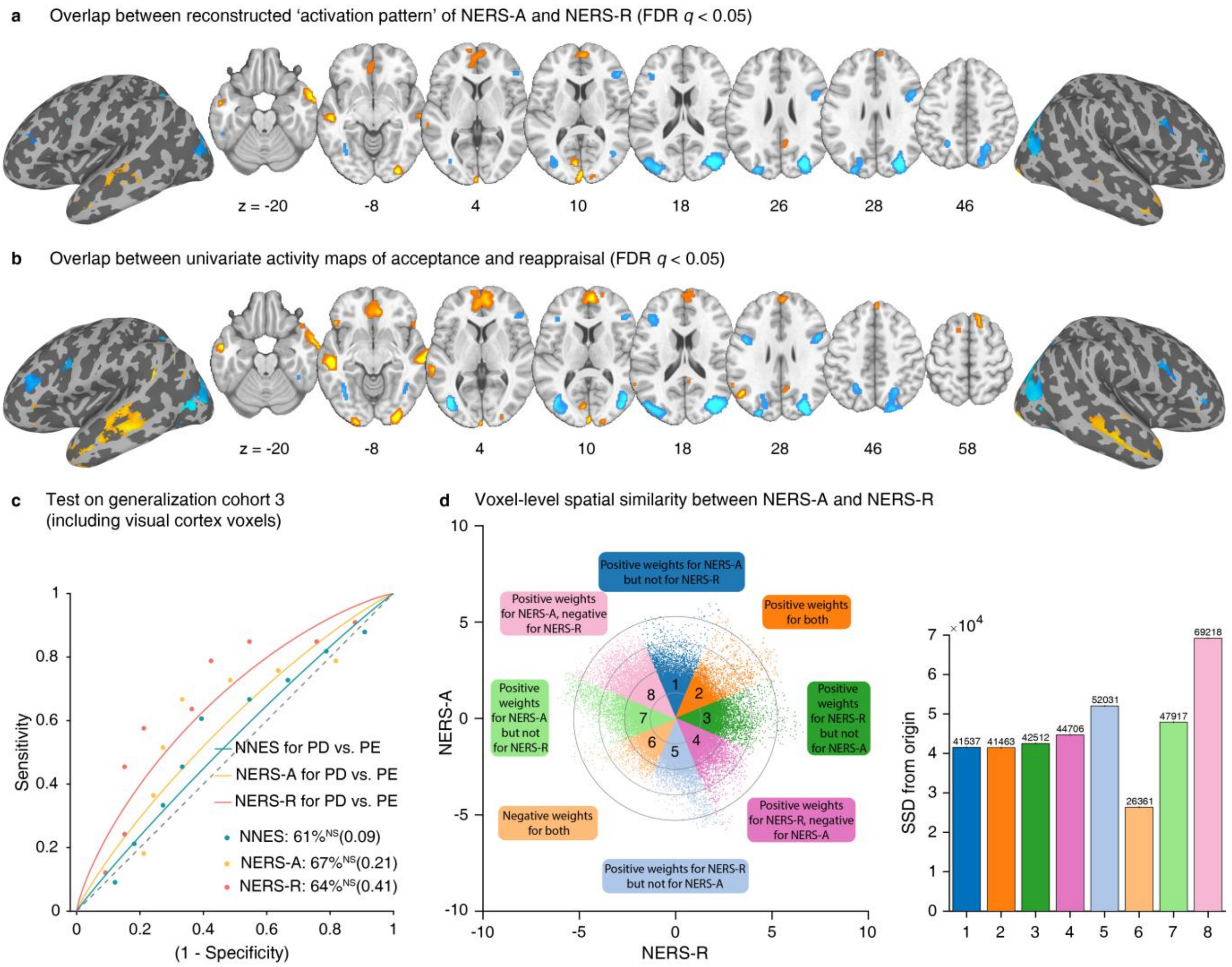

Fig.S4 | Common brain regions identified by conjunction analysis, predictive performance on generalization cohort 3 (including visual cortex voxels), and voxel-level spatial similarity between NERS-A and NERS-R. The conjunction of the FDR corrected (q<0.05) reconstructed 'activation patterns' between NERS-A and NERS-R (**a**) as well as univariate activity maps between NA vs. NV and NR vs. NV (**b**). **c**, The classification performances of the developed decoders on the generalization cohort 3 (n=33)[4], including voxels from the visual cortex. P_{NNES}=0.296; $P_{\text{NERS-A}}$=0.080; $P_{\text{NERS-R}}$=0.163. **d**, Voxel-level spatial similarity between the decoding weighted map of NERS-A and NERS-R. Scatter plots illustrate normalized unthresholded voxel beta weights of the NERS-A (y-axis) and NERS-R (x-axis). Colored octants indicate voxels of shared positive (Octants 2) or shared negative (Octants 6), selective positive weights

for NERS-A (Octant 1) or NERS-R (Octant 3), selective negative weights for NERS-A (Octant 5) or NERS-R (Octant 7), and voxel weights opposite for the two signatures (Octants 4 and 8). The right bars indicate the sum of squared distances from the origin (0, 0) for each octant, which integrates the number of voxels and combined weights. PD, down-regulate heat-induced pain; PE, experience heat-induced pain.

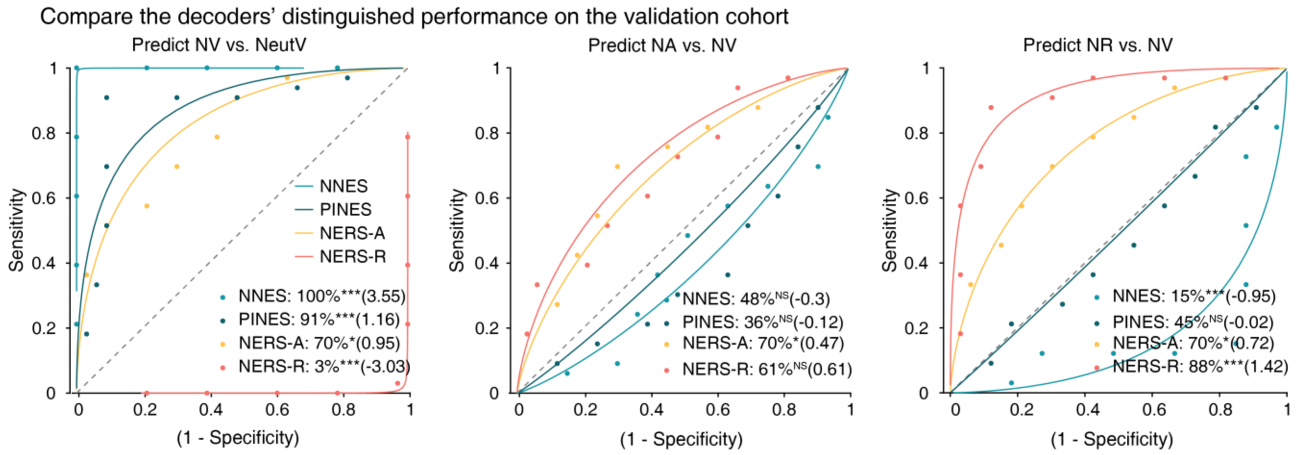

Fig.S5 | Comparison of decoders' predictive performance. Forced-choice classification accuracy and Cohen's d indicate that the NNES, NERS-A, and NERS-R could accurately predict the targeted mental processing during discrimination at NV vs. NeutV, NA vs. NV (left), and NR vs. NV (middle), respectively, based on the validation cohort. PINES could distinguish NV vs. NeutV but not ER strategies vs. NV. Detailed statistical results are provided in Table S2.

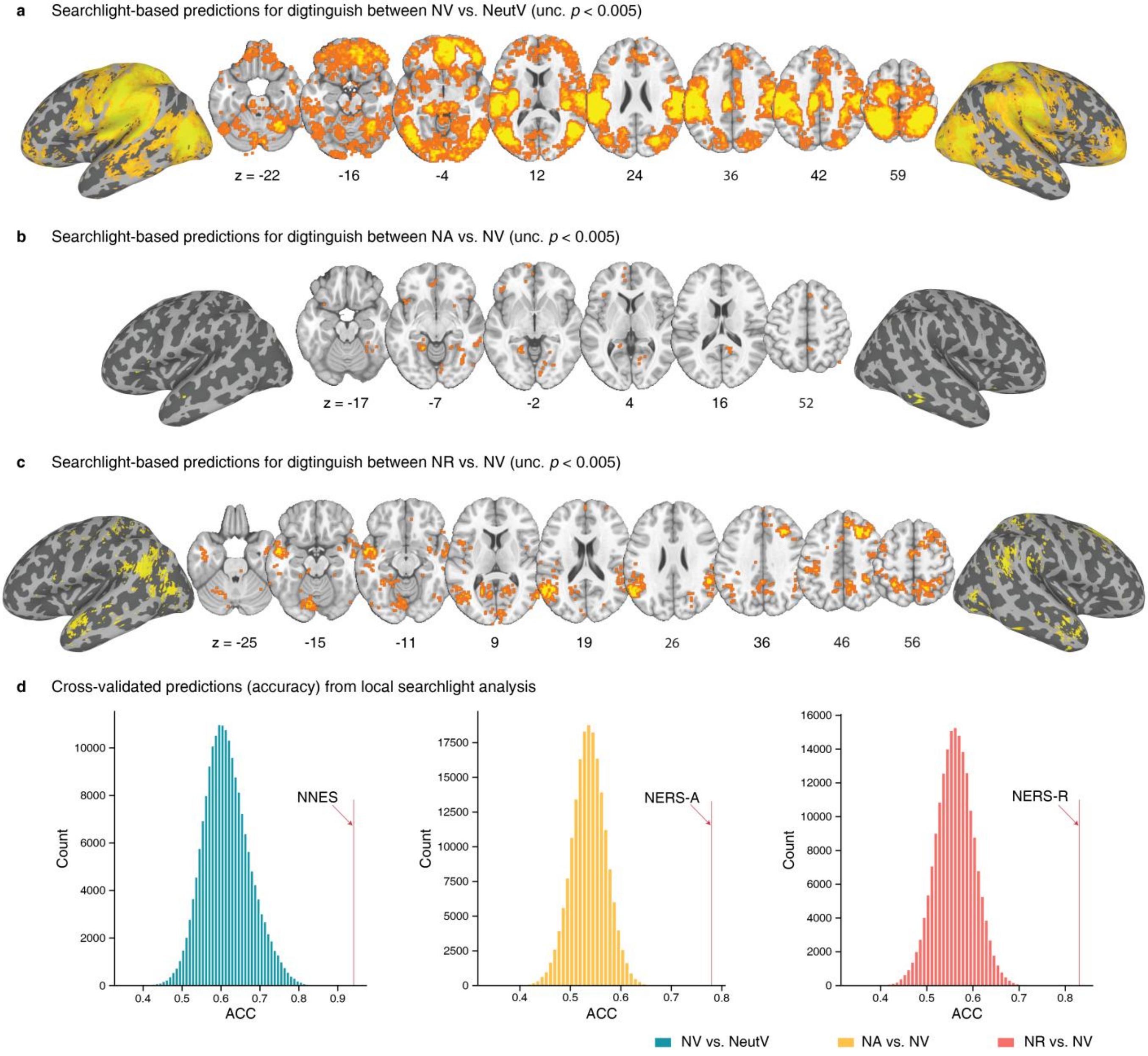

Fig.S6 | Searchlight-based predictions in the discovery cohort. Brain regions that significantly predict the response (**a**), acceptance (**b**), and reappraisal (**c**) to naturalistic stimuli that are revealed by searchlight-based analyses. **d**, Histograms: cross-validated predictions from local searchlights. ACC, accuracy.

References

1. Zimmermann, K. *et al.* Emotion regulation deficits in regular marijuana users. *Hum. Brain Mapp.* **38**, 4270–4279 (2017).
2. Sheehan, D. V. The Mini-International Neuropsychiatric Interview (M.I.N.I.): The Development and Validation of a Structured Diagnostic Psychiatric Interview for DSM-IV and ICD-10.
3. Lang, P. J., Bradley, M. M. & Cuthbert, B. N. International affective picture system (IAPS): Technical manual and affective ratings. *NIMH Cent. Study Emot. Atten.* 39–58 (1997).
4. Woo, C.-W., Roy, M., Buhle, J. T. & Wager, T. D. Distinct Brain Systems Mediate the Effects of Nociceptive Input and Self-Regulation on Pain. *PLoS Biol.* **13**, e1002036 (2015).
