# Supplementary material for: Common and distinct neurofunctional signatures of dynamic naturalistic emotion regulation strategies
Source: Nat Commun. 2026 Mar 17;17:4272. doi: 10.1038/s41467-026-70708-5 (PMC13168294; doi:10.1038/s41467-026-70708-5)
Supplement: Supplementary file 2 — Reporting Summary [file 41467_2026_70708_MOESM2_ESM.pdf]

nature portfolio

Corresponding author(s): Feng Zhou, Benjamin Becker

Last updated by author(s): Feb 19, 2026

# Reporting Summary

Nature Portfolio wishes to improve the reproducibility of the work that we publish. This form provides structure for consistency and transparency in reporting. For further information on Nature Portfolio policies, see our Editorial Policies and the Editorial Policy Checklist.

## Statistics

For all statistical analyses, confirm that the following items are present in the figure legend, table legend, main text, or Methods section.

| n/a | Confirmed | |
|---|---|---|
| ☐ | ☒ | The exact sample size (*n*) for each experimental group/condition, given as a discrete number and unit of measurement |
| ☐ | ☒ | A statement on whether measurements were taken from distinct samples or whether the same sample was measured repeatedly |
| ☐ | ☒ | The statistical test(s) used AND whether they are one- or two-sided<br>*Only common tests should be described solely by name; describe more complex techniques in the Methods section.* |
| ☐ | ☒ | A description of all covariates tested |
| ☐ | ☒ | A description of any assumptions or corrections, such as tests of normality and adjustment for multiple comparisons |
| ☐ | ☒ | A full description of the statistical parameters including central tendency (e.g. means) or other basic estimates (e.g. regression coefficient) AND variation (e.g. standard deviation) or associated estimates of uncertainty (e.g. confidence intervals) |
| ☐ | ☒ | For null hypothesis testing, the test statistic (e.g. *F*, *t*, *r*) with confidence intervals, effect sizes, degrees of freedom and *P* value noted<br>*Give P values as exact values whenever suitable.* |
| ☐ | ☒ | For Bayesian analysis, information on the choice of priors and Markov chain Monte Carlo settings |
| ☒ | ☐ | For hierarchical and complex designs, identification of the appropriate level for tests and full reporting of outcomes |
| ☐ | ☒ | Estimates of effect sizes (e.g. Cohen's *d*, Pearson's *r*), indicating how they were calculated |

*Our web collection on statistics for biologists contains articles on many of the points above.*

## Software and code

Policy information about availability of computer code

| | |
|---|---|
| Data collection | Stimuli in the discovery and validation cohorts were presented using the E-Prime software (Version 3.0; Psychology Software Tools, Sharpsburg, PA), and stimuli in the clinical application cohort were presented using Presentation 14.9 (Neurobehavioral Systems Inc., Albany, CA, USA). For data provided by previous studies, please refer to the original publications. |
| Data analysis | Preprocessing, first-level, and second-level modelings of fMRI data were analyzed using SPM12 (https://www.fil.ion.ucl.ac.uk/spm/software/spm12), Multivariate pattern analysis was conducted using the CANLabCore toolbox (https://github.com/canlab/CanlabCore). The above analysis was implemented in MATLA 2023b. Custom codes used in this study are available at https://github.com/h-psy/fMRI_studies/tree/main/NERS.<br>Code for analyzing data and generating figures is available via GitHub at https://github.com/canlab and https://github.com/h-psy/fMRI_studies/tree/main/NERS. A version of the code used in this study is available via Zenodo (https://doi.org/10.5281/zenodo.18625297). |

For manuscripts utilizing custom algorithms or software that are central to the research but not yet described in published literature, software must be made available to editors and reviewers. We strongly encourage code deposition in a community repository (e.g. GitHub). See the Nature Portfolio guidelines for submitting code & software for further information.

## Data

Policy information about availability of data

All manuscripts must include a data availability statement. This statement should provide the following information, where applicable:

- Accession codes, unique identifiers, or web links for publicly available datasets
- A description of any restrictions on data availability
- For clinical datasets or third party data, please ensure that the statement adheres to our policy

fMRI data used to train the signatures are available via figshare at https://doi.org/10.6084/m9.figshare.29179934.v1. Other authors provided the fMRI data in the generalization cohort 1 via NeuroVault (https://neurovault.org/collections/16266), and fMRI data in the generalization cohort 2 via the OpenfMRI (https://openfmri.org/dataset/ds000140).

## Research involving human participants, their data, or biological material

Policy information about studies with human participants or human data. See also policy information about sex, gender (identity/presentation), and sexual orientation and race, ethnicity and racism.

| | |
|---|---|
| Reporting on sex and gender | We confirm that we use the terms sex and gender in ways consistent with their definitions. This project included both male and female participants, and the gender was determined based on self-report, in this project we did not perform specific gender-based analysis because our main aim was to identify sensitive and robust neural signatures for using emotion regulation strategies (acceptance and reappraisal) and experience of naturalistic negative emotions that across all humans. |
| Reporting on race, ethnicity, or other socially relevant groupings | The project did not collect information about race and ethnicity from the participants. |
| Population characteristics | Discovery cohort: n = 59 participants (19 females; mean±SD age = 21.3±1.7 years old); Validation cohort: n= 33 participants (11 females; 20.3±1.4 years old); Clinical application cohort: all male participants, n_cannabis_users: 49 participants (24.1 ±5.3 years old), n_healthy_controls: 48 participants (24.3±5.0 years old). For data provided by previous studies, please refer to the original publications. |
| Recruitment | Healthy volunteers were recruited by means of local advertisements, Exclusion criteria included color blindness: current or regular substance or medication use, current or history of medical or psychiatric disorders; and any contraindications for MRI. We do not expect self-selection biases to substantially influence findings. For the cannabis users and corresponding healthy control participants, please see supplementary for the inclusion/exclusion criteria. |
| Ethics oversight | The study of the discovery and validation cohort was approved by the ethics committee of the University of Electronic Science and Technology of China. The study of the clinical application cohort was approved by the ethics committee of the University of Bonn, Bonn, Germany. For data provided by previous studies, please refer to the original publications. |

Note that full information on the approval of the study protocol must also be provided in the manuscript.

# Field-specific reporting

Please select the one below that is the best fit for your research. If you are not sure, read the appropriate sections before making your selection.

☒ Life sciences ☐ Behavioural & social sciences ☐ Ecological, evolutionary & environmental sciences

For a reference copy of the document with all sections, see nature.com/documents/nr-reporting-summary-flat.pdf

# Life sciences study design

All studies must disclose on these points even when the disclosure is negative.

| | |
|---|---|
| Sample size | No statistical tests were used to predetermine the sample size, but this sample size is within the standard range and in accordance with the suggestions in the field (see e.g., Kohoutova et al., 2020; Zhou et al., 2021). To further validate the robustness of the findings independent one validation cohort, two generalization cohorts, and a large independent clinical dataset were included. |
| Data exclusions | Discovery cohort: data from nine participants were excluded due to excessive head movement (>3mm) during the fMRI scanning or the post-check; validation cohort: data from one participant was excluded due to being detected as an outlier; clinical application cohort: three HC and seven CU newly collected participants were excluded due to excessive head motion; three HC and three CU were excluded due to being detected as outliers based on their emotion regulation success. |
| Replication | Cross-validation (and testing in another four independent cohorts) showed that the effects were replicated across samples, MRI systems, culture, and stimuli. |
| Randomization | Not applicable. The current study does not include intervention/placebo groups, thus no randomization was performed. |
| Blinding | Not applicable. |

# Reporting for specific materials, systems and methods

We require information from authors about some types of materials, experimental systems and methods used in many studies. Here, indicate whether each material, system or method listed is relevant to your study. If you are not sure if a list item applies to your research, read the appropriate section before selecting a response.

## Materials & experimental systems

| n/a | Involved in the study |
| --- | --- |
| ☒ | ☐ Antibodies |
| ☒ | ☐ Eukaryotic cell lines |
| ☒ | ☐ Palaeontology and archaeology |
| ☒ | ☐ Animals and other organisms |
| ☒ | ☐ Clinical data |
| ☒ | ☐ Dual use research of concern |
| ☒ | ☐ Plants |

## Methods

| n/a | Involved in the study |
| --- | --- |
| ☒ | ☐ ChIP-seq |
| ☒ | ☐ Flow cytometry |
| ☐ | ☒ MRI-based neuroimaging |

## Plants

| | |
| --- | --- |
| Seed stocks | Not applicable. |
| Novel plant genotypes | Not applicable. |
| Authentication | Not applicable. |

## Magnetic resonance imaging

### Experimental design

| | |
| --- | --- |
| Design type | Task-based fMRI |
| Design specifications | Discovery and Validation Cohorts: Each cohort completed 48 trials distributed across 4 runs. Each trial began with a 3–5 second fixation, followed by a 2-second cue indicating the condition (react, acceptance, or reappraisal). This was followed by another 6–8 second fixation period, then a 25-second video clip. After the video, a 1–1.5 second fixation was shown, followed by a 4-second rating period. At the end of each run, participants were shown a scale for 4 seconds to rate their average level of success in that run. Both the discovery and validation cohorts were pre-registered (discovery cohort: https://osf.io/s3bp2; validation cohort: https://osf.io/68jkw). The newly collected data in the clinical application cohort followed a similar procedure to our previous study (Zimmermann et al., 2017). For further details, please refer to the Supplementary Materials and our earlier publication. |
| Behavioral performance measures | Subjective negative feeling rating. |

### Acquisition

| | |
| --- | --- |
| Imaging type(s) | Functional |
| Field strength | 3T |
| Sequence & imaging parameters | Functional images were acquired using a T2*-weighted echo planar imaging (EPI) sequence. Detailed acquisition parameters for the discovery cohort were: repetition time (TR) = 2000 ms, echo time (TE) = 30 ms, flip angle (FA) = 80°, matrix size = 80 × 80, voxel size = 3 × 3 × 3.2 mm, 34 interleaved ascending axial slices, with phase encoding in the posterior-to-anterior direction. For the validation cohort, parameters were: TR = 2000 ms, TE = 29 ms, field of view (FOV) = 240 mm, FA = 90°, matrix size = 80 × 80, voxel size = 3 × 3 × 3 mm, 36 interleaved ascending axial slices, also with posterior-to-anterior phase encoding. For acquisition details of other cohorts, please refer to the original publications. |
| Area of acquisition | Whole brain. |

Diffusion MRI ☐ Used ☒ Not used

## Preprocessing

| | |
|---|---|
| Preprocessing software | SPM12 |
| Normalization | Nonlinear deformation based on anatomical data. |
| Normalization template | ICBM152 space |
| Noise and artifact removal | First-level models included motion parameter estimates. Nuisance variables encompassed: (1) 'dummy' regressors representing each run (intercept for each run); (2) the six estimated head movement parameters (X, Y, Z, roll, yaw, and pitch), their squares, their derivatives and squared derivatives for each run (24 columns in total); and (3) vectors indicating motion outlier time points. |
| Volume censoring | No censoring, instead inclusion of motion spikes as regressors in the first-level model |

## Statistical modeling & inference

| | |
|---|---|
| Model type and settings | First-level models were mass-univariate, and second-level analyses included both univariate and multivariate models. |
| Effect(s) tested | Linear parametric effects of negative emotional experience and its regulation via acceptance or reappraisal;  multivariate pattern based classification and representational similarity analysis. |

Specify type of analysis: ☒ Whole brain ☐ ROI-based ☐ Both

| | |
|---|---|
| Statistic type for inference (See Eklund et al. 2016) | Voxel-wise |
| Correction | FDR correction |

## Models & analysis

| n/a | Involved in the study |
|---|---|
| ☒ | ☐ Functional and/or effective connectivity |
| ☒ | ☐ Graph analysis |
| ☐ | ☒ Multivariate modeling or predictive analysis |

| | |
|---|---|
| Multivariate modeling and predictive analysis | The support vector machine (SVM) model was used to predict the corresponding mental process—experiencing, accepting, or reappraising negative emotions elicited by naturalistic video clips—versus the respective control condition. Model features included neural activation patterns associated with each mental process. Prediction performance and effect sizes (Cohen's d) were evaluated using both cross-validation and independent sample validation. |
